# A multiple myeloma-specific capture sequencing platform discovers novel translocations and frequent, risk-associated point mutations in *IGLL5*

**DOI:** 10.1038/s41408-018-0062-y

**Published:** 2018-03-21

**Authors:** Brian S. White, Irena Lanc, Julie O’Neal, Harshath Gupta, Robert S. Fulton, Heather Schmidt, Catrina Fronick, Edward A. Belter, Mark Fiala, Justin King, Greg J. Ahmann, Mary DeRome, Elaine R. Mardis, Ravi Vij, John F. DiPersio, Joan Levy, Daniel Auclair, Michael H. Tomasson

**Affiliations:** 1 Department of Medicine, Washington University School of Medicine, St. Louis, 63110 MO USA; 2 McDonnell Genome Institute, Washington University School of Medicine, St. Louis, 63108 MO USA; 3 Division of Hematology-Oncology, Mayo Clinic, Rochester, 55905 MN USA; 4 Multiple Myeloma Research Foundation, Norwalk, CT 06851 USA; 5 Present Address: Sage Bionetworks, Seattle, WA 91809 USA; 6 Present Address: Genomics Institute, Nationwide Children’s Hospital, Columbus, OH 43205 USA; 7 Present Address: Division of Hematology, Oncology and Bone Marrow Transplantation, 5204 MERF, University of Iowa, Iowa City, IA 52242 USA; 8 Present Address: Chordoma Foundation, Durham, NC 27702 USA

## Abstract

Multiple myeloma (MM) is a disease of copy number variants (CNVs), chromosomal translocations, and single-nucleotide variants (SNVs). To enable integrative studies across these diverse mutation types, we developed a capture-based sequencing platform to detect their occurrence in 465 genes altered in MM and used it to sequence 95 primary tumor-normal pairs to a mean depth of 104×. We detected cases of hyperdiploidy (23%), deletions of 1p (8%), 6q (21%), 8p (17%), 14q (16%), 16q (22%), and 17p (4%), and amplification of 1q (19%). We also detected *IGH* and *MYC* translocations near expected frequencies and non-silent SNVs in *NRAS* (24%), *KRAS* (21%), *FAM46C* (17%), *TP53* (9%), *DIS3* (9%), and *BRAF* (3%). We discovered frequent mutations in *IGLL5* (18%) that were mutually exclusive of *RAS* mutations and associated with increased risk of disease progression (*p* = 0.03), suggesting that *IGLL5* may be a stratifying biomarker. We identified novel *IGLL5*/*IGH* translocations in two samples. We subjected 15 of the pairs to ultra-deep sequencing (1259×) and found that although depth correlated with number of mutations detected (*p* = 0.001), depth past ~300× added little. The platform provides cost-effective genomic analysis for research and may be useful in individualizing treatment decisions in clinical settings.

## Introduction

Multiple myeloma (MM) is a fatal malignancy of mature plasma B cells. Overt MM is preceded by a premalignant phase, monoclonal gammopathy of undetermined significance (MGUS), which can progress to smoldering MM and ultimately to fatal myeloma. Genetic alterations detected in premalignant MGUS cells are likely initiating events. These may be divided into two primary subtypes that are most often non-overlapping^1^: hyperdiploid (HRD) myeloma is characterized by trisomies of most odd-numbered chromosomes^2^, while non-HRD myeloma frequently involves immunoglobulin heavy chain (*IGH*) translocations. These upregulate target oncogenes by placing them under the control of one or both of the powerful, B-cell-specific *IGH* enhancer regions; canonical *IGH* partner genes include *WHSC1*/*FGFR3*, *CCND3*, *CCND1*, *MAF*, and *MAFB* in translocations t(4;14), t(6;14), t(11;14), t(14;16), and t(14;20), respectively^1^. Secondary genetic events are detected in MM, but not its precursor phases, and are thought to drive disease progression. The most prevalent secondary events include *MYC* translocations (juxtaposing *IGH* and other loci), single-nucleotide variants (SNVs) involving *KRAS*, *NRAS*, and *DIS3*, and copy number variants (CNVs) that amplify chromosome arm 1q or delete 1p, 6q, 13, 14q, or 16q^2^.

This diversity of genetic lesions has recently been leveraged in a prognostic model that integrates the International Staging System (ISS)^3^ with incidence of CNVs, SNVs, and translocations^4^. This ISS-MUT model increases precision over ISS alone in detecting early mortality and progression. Other studies have highlighted the context-dependent prognostic significance of variants across the spectrum of mutation types^1^. For example, trisomies of chromosomes 3 or 5 have been found to abrogate the poor overall survival associated with t(4;14) translocations^5^. Collectively, these results highlight the prognostic impact of the interplay between CNVs, SNVs, and translocations—the potential for co-occurring mutations to mitigate an otherwise poor outcome implies that testing a patient for all three types of mutations may help target chemotherapy regimens more accurately to specific patient subgroups in the future^2^.

Detecting myeloma-relevant mutations may be done via exome sequencing, as in the ISS-MUT study^4^. However, approaches targeting a subset of disease-associated genes should reduce computational analysis, facilitate quicker return of clinical results, and enable deeper sequencing at a fixed budget^6^. Indeed, targeted, clinical sequencing is performed with increasing frequency both commercially^7^ and through cancer centers^8–10^. In the specific context of MM, an amplicon-based, 77-gene panel detects both CNVs and SNVs^11^. This extends an earlier panel^6^ used to track mutation evolution across 47 genes^12^. Other efforts have focused on *IGH* rearrangements and translocations: amplicon-based sequencing of the locus effectively detects minimal residual disease^13^, while capture-based approaches have been used to discover *IGH* and *MYC* translocations^14,15^.

Similar enrichment technologies could be used to simultaneously detect CNVs, SNVs, and translocations. Indeed, one such platform was recently described—the approach involved targeted sequencing of the *IGH* locus and 246 genes implicated in MM and/or other cancers^16^. The platform was used to profile 14 MM cell lines and five primary samples. Maturing such approaches will require extensive validation and tuning of the associated bioinformatics methods. Towards this end, we developed a related MM-specific targeted sequencing platform and validated it against 95 primary tumor samples, 44 of which were previously subjected to exome sequencing and 22 of which were previously assayed by fluorescence in situ hybridization (FISH). We demonstrate high concordance of the platform with these exome sequencing and FISH results. Additionally, we describe novel approaches to tuning computational CNV and translocation calling methods. These optimized methods facilitated integrative analysis across mutation types, which revealed patterns of mutual exclusivity and co-occurrence involving CNVs, SNVs, and translocations. We discovered novel translocations juxtaposing *IGLL5* with *IGH* and detected high-frequency mutations in *IGLL5*. These *IGLL5* mutations were mutually exclusive of *RAS* mutations and associated with disease progression, suggesting that *IGLL5* may be involved in disease pathogenesis and/or serve as a biomarker of high-risk MM.

## Methods

We designed a Nimblegen probe set (Roche) targeting 3.3 Mb of space that includes 465 genes and the *IGH* region. Sequencing library pools were prepared, hybridized to the probes, and sequenced on the HiSeq2000 (2 × 100 reads for initial sequencing of 95 tumor-normal pairs) or the HiSeq2500 (2 × 125 reads for deep sequencing of a subset of 15 pairs). Reads were aligned against human reference genome GRCh37-lite using BWA^17^. Acquired SNVs were called in each tumor sample relative to its paired normal sample using samtools^18^, SomaticSniper^19^, MuTect^20^, Strelka^21^, and VarScan2 (ref. ^22^). Translocations were called using LUMPY^23^, with results filtered by a machine learning approach optimized to achieve high precision relative to available FISH results. CNVs were called using CopyCAT2 (https://github.com/abelhj/cc2/; ref.^24^) parameterized to detect copy number alterations exceeding the level of noise estimated from diploid regions using a gaussian mixture model (https://github.com/genome/bmm). Additional information is provided in Supplementary Methods.

## Results

We developed a MM-specific custom capture sequencing platform capable of detecting CNVs, SNVs, and translocations. We designed oligonucleotide probes covering 3.3 Mb of genomic space and complementary to the exons, untranslated regions, and splice sites of 465 genes (Tables S1 and S2) expressed in MM that: (1) are annotated as cancer genes (in COSMIC^25^ or MutSig^26^), (2) function in DNA repair or B-cell biology, (3) are mutated at a frequency of >3% in published studies^27,28^, or (4) have mutations that cluster in hotspots. To detect *IGH* translocations, we also designed probes tiled in an unbiased manner across the locus, including within the variable (*IGHV*), diversity (*IGHD*), joining (*IGHJ*), and constant/switch regions. We also designed probes targeting the exonic regions of canonical *IGH* translocation partners (*CCND1*, *CCND3*, *FGFR3*, *MAF*, *MAFB*, *WHSC1*, and *WWOX*). To capture secondary *MYC* translocations, we tiled probes across exonic and intronic regions of the *MYC* locus.

We used the platform to sequence DNA isolated from 95 tumor (CD138-purified cells isolated from bone marrow aspirates) and paired normal (blood) samples. These samples were specifically selected to validate our platform and to tune our computational methods as a subset of them were previously subjected to exome sequencing (44 samples) and/or FISH (22 samples) analysis of *IGH* translocations. We achieved a mean sequencing depth of 104× (min = 33×, max = 140×; Supplementary Figure S1A) across the tumor samples and of 107× (min = 43×, max = 168×; Supplementary Figure S1B) across the normal samples.

### Targeted capture sequencing identifies copy number alterations with prognostic significance

**Fig. 1:**
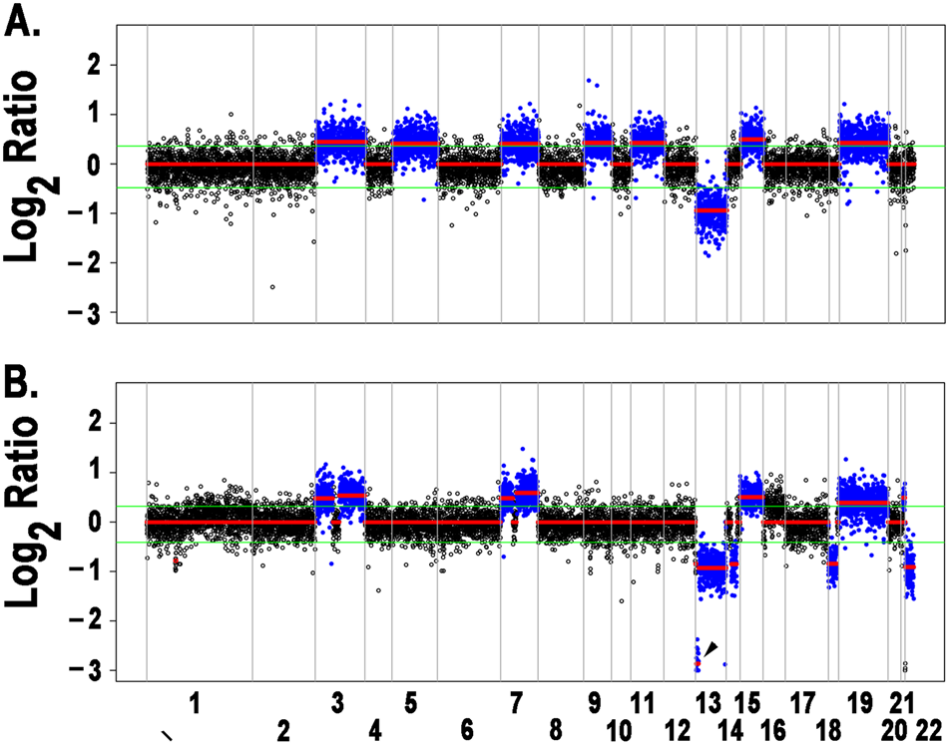
Targeted sequencing identifies chromosome-level, arm-level, and focal CNVs. **a** Hyperdiploid and (**b**; arrow) focal copy number events detected by CopyCat2 (blue; *p* < 0.05) from log_2_ ratios of tumor to paired normal sequencing depth (*y*-axis) across chromosomes (*x*-axis). (Clonal) single-copy gains occur at a log_2_ ratio of log_2_(3/2)  =0.58, whereas (clonal) heterozygous/single-copy losses occur at a log_2_ ratio of log_2_(1/2) = −1

The broad coverage of the platform [across chromosome arms (Table S3) and 465 genes] facilitated detection of chromosome-level, arm-level, and focal CNVs. We identified these events computationally from the per-probe ratios of tumor to normal sequencing depth using CopyCAT2 (ref.^24^). To reduce false positives, we developed an approach that filtered CNV calls with ratios below a noise level estimated from diploid regions (Supplementary Figure S2A–C). Our approach identified the full range of CNVs, from genome-scale hyperdiploid events (Fig. 1a; Table S4) to focal events, including a homozygous deletion that encompassed *BRCA2* (Fig. 1b). Detected arm-level events included those associated with poor prognosis such as amp(1q), del(1p), del(13q), and del(17p) (reviewed in ref.^1^; Supplementary Figure S2D).

### Targeted capture sequencing identifies *IGH* translocations

**Fig. 2:**
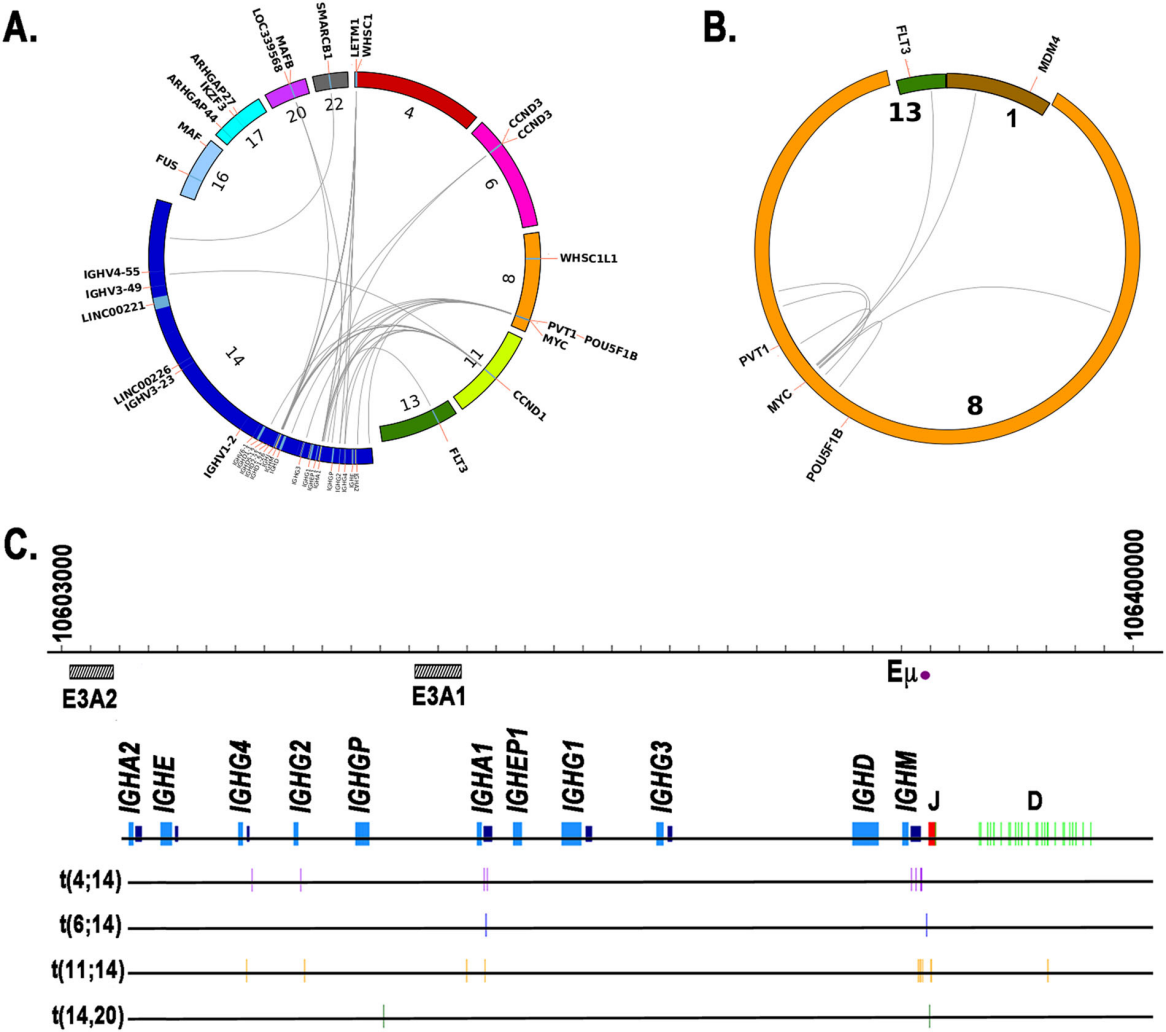
Targeted sequencing detects *IGH* and *MYC* translocations. Circos plots of (**a**) *IGH* and (**b**) *MYC* translocations. Chromosomes involved in translocations are magnified to highlight regions and genes near breakpoints. **c** Breakpoints (vertical lines) of canonical *IGH* translocations within *IGH* locus. E3A2 and E3A1: 3’ enhancer elements downstream of *IGHA2* and *IGHA1* genes, respectively. Eμ: μ enhancer. Purple boxes: switch regions. Figure is to scale

We detected *IGH* translocations using LUMPY and again developed a filtering strategy to reduce likely false positives. We filtered putative translocations based on thresholds on the number of supporting split reads and discordant paired-end reads. We tuned the thresholds to maximize precision using a machine learning approach and available FISH data, resulting in a precision of 100% and a recall of 64% (Supplementary Figure S3; Supplementary Table S5). Canonical *IGH* translocations were then detected by the platform near expected frequencies (Table 1; Fig. 2a) and occurred predominantly within the *IGH* constant region, but also telomeric of the *IGHM* switch region and occasionally within the D and J regions (Fig. 2c). Notably, one of the t(11;14) translocations occurred within the constant region, but outside all constant and switch segments. These results are consistent with a recent study^15^, which found *IGH* translocations occurring within each of these regions. No translocations within the V region passed the filtering step.

**Table 1:** Frequency of Translocations

Transloaction	Frequency (%)
t(11;14)	14
t(4;14)	13
t(8;14)	7
t(6;14)	2
t(14;20)	2

### *IGLL5* is translocated and co-incident with overexpression of *DERL3* in multiple myeloma

To prioritize novel *IGH* translocations as potential driver mutations, we identified cancer-associated genes within 1 Mb of each chromosomal breakpoint (Supplementary Table S6). The two annotated translocations with largest total evidence (sum of number of supporting split reads and number of discordant paired-end reads) were analyzed further. The first was a complex translocation involving chromosomes 11, 13, and 14. The putative breakpoint on chromosome 13 was nearby *FLT3* (<0.5 Mb; Supplementary Figure S4); we validated that chromosome 13q12.2 was indeed translocated to *IGH* on chromosome 14 using PCR.

**Fig. 3:**
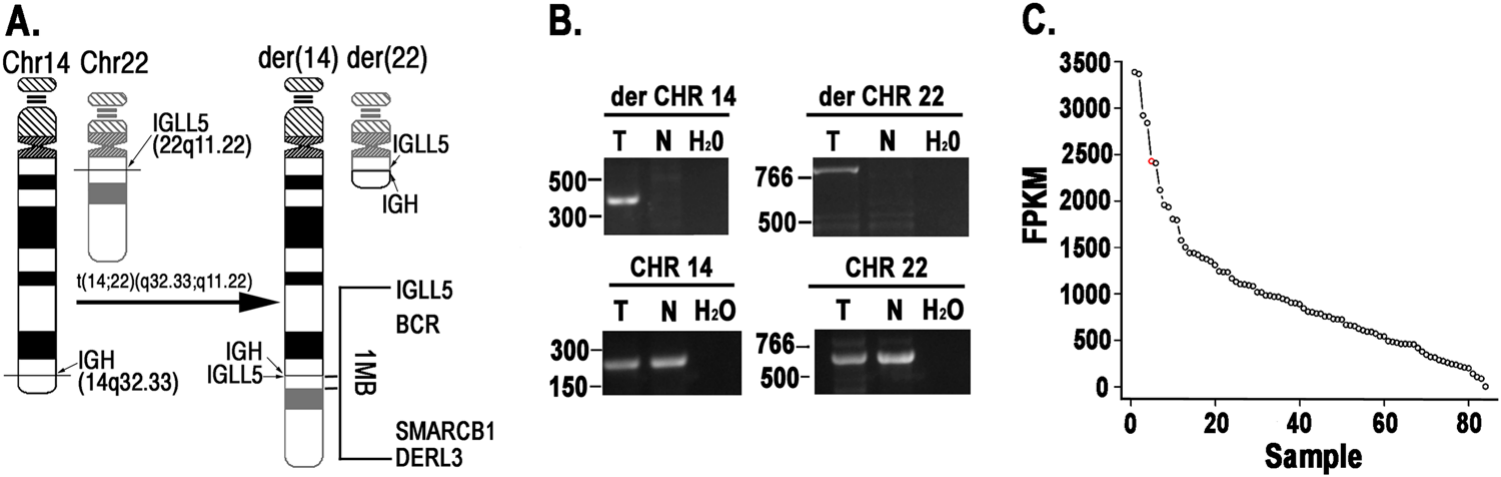
*IGLL5* is translocated and *DERL3* is overexpressed in multiple myeloma. **a** Schematic of validated t(14;22) translocation. (Left) WT chromosomes 14 and 22 with horizontal lines indicating location of breakpoints within the *IGH* and *IGLL5* loci, respectively. (Right) Two derivative (der) chromosomes, each retaining a portion of its respective *IGH* or *IGLL5* gene. Cancer-associated genes within 1 Mb of breakpoint on der(14) are shown. **b** PCR validation of t(14,22) translocation. Oligos specific to each breakpoint used in PCR reactions (top). Oligos specific to the small regions deleted on der(14) and der(22) were designed to detect non-translocated allele (bottom). T tumor, N germline (peripheral blood) control. **c***DERL3* expression across 84 MM patients. Red circle indicates sample in which putative (non-validated) t(14;22) translocation was detected. FPKM Fragments Per Kilobase of transcript per Million mapped reads

Breakpoints of the second highly-supported translocation, t(14,22)(q32.33;q11.22), were located within *IGH* and *IGLL5*, which is spanned by the immunoglobulin lambda light chain locus (Fig. 3a). To validate this translocation, we performed PCR amplification of the putative breakpoint on DNA isolated from the patient in which it was detected. A PCR product of the expected size was detected in CD138^+^ tumor cells but not in the peripheral blood mononuclear control [Fig. 3b (top)]. Re-sequencing and mapping of the tumor-specific PCR product confirmed the reciprocal translocation spanned chromosomes 14 and 22. Small regions were deleted on both derivative chromosomes and thus could be used to selectively amplify the corresponding wild-type (WT) chromosomes. We designed primers within these deleted regions and used them to perform PCR amplification, which confirmed the retention of one copy of each of the WT chromosomes in the tumor sample [Fig. 3b (bottom)].

To search for additional *IGLL5* translocations, we relaxed our filtering constraints and found a second sample predicted by LUMPY to harbor a t(14;22) translocation, though no DNA was available for validation. The validated t(14;22) translocation was predicted to juxtapose the μ and 3’ enhancers (chr14:106032614–106167601) with *DERL3*. Hence, we looked for evidence of overexpression of *DERL3* and other cancer-associated genes within 1 Mb of the predicted breakpoint on chromosome 22 (*IGLL5, BCR*, and *SMARCB1*) by examining RNA-seq expression data from a partially overlapping set of 84 MM patients. We found outlying expression of *DERL3* in six of these samples (exceeding 1.5× the FPKM interquartile range), including the second sample with a putative t(14;22) translocation (Fig. 3c; no expression data were available for the sample harboring the validated translocation). Additionally, we found that *DERL3* was overexpressed in MM relative to other cancer types within the Cancer Cell Line Encyclopedia (CCLE)^29^ (Supplementary Figure S5), with high expression also reported in other B-cell malignancies. We also examined expression of *IGLL5* both within our cohort and CCLE. *IGLL5* expression was in the 82nd percentile in the putatively translocated sample for which we had RNA-seq data, though it is not noticeably elevated with respect to the mean expression (data not shown). *IGLL5* expression in MM cell lines within the CCLE is second only to Burkitt’s Lymphoma cell lines; more generally, and as with *DERL3*, *IGLL5* expression is higher in B-cell malignancies than in other cancer types. Taken together, these data suggest that *DERL3* may be dysregulated in MM via *IGH* translocation or another unknown mechanism.

### Targeted capture sequencing identifies intra- and inter-chromosomal *MYC* translocations

FISH validation data of *MYC* translocations were not available to tune LUMPY parameters and, as a result, intra- and (non-*IGH*) inter-chromosomal *MYC* translocations were called at a high false-positive rate (in every tumor and normal sample, Supplementary Figure S6). To accurately detect somatic *MYC* translocations, we developed a machine learning-based approach tuned to filter putative *MYC* translocations called in normal samples. Applying this method to tumor samples resulted in five intra-chromosomal and two non-*IGH* inter-chromosomal *MYC* translocations, with one sample having one intra- and one inter-chromosomal translocation (6 of 95, 6%, Fig. 2b; Supplementary Table S7). The intra-chromosomal translocations involved neighboring genes *PVT1* and *POU5F1B*, as previously reported^14^.

### Targeted capture sequencing identifies non-silent single-nucleotide variants in all tumor samples

All tumor samples harbored at least one somatic (missense, nonsense, or frame-shift) mutation, with each sample having a mean of 20 mutations (Supplementary Table S8). A total of 443 genes had a non-synonymous (frame-shift insertion or deletion, missense, or nonsense) mutation in one or more samples; 581 genes had a mutation of any kind in one or more samples. Ninety-four of 95 tumor samples had a mutation predicted to be deleterious by Poly-Phen2^30^ or SIFT^31^, with each sample having a mean of twelve deleterious mutations. In 24 instances, we observed a gene harboring multiple mutations previously associated with cancer (via COSMIC). This occurred in thirteen samples across seventeen genes, including *KRAS* and *RB1*; both were among the most frequently observed (in three samples).

### Increased sequencing depth yields few additional variants

**Fig. 4:**
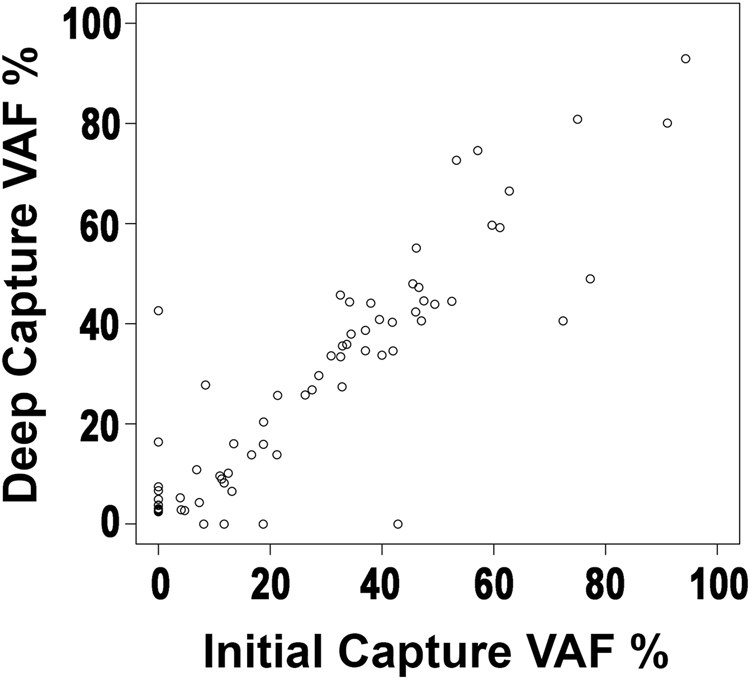
Greater sequencing depth yields few additional variants. VAF of variants discovered during initial targeted sequencing (*x*-axis) and/or with subsequent deeper sequencing (*y*-axis)

To determine whether MM is characterized by deeply subclonal variants of biological significance, we performed additional sequencing of 15 tumor (mean depth = 1,259×, min = 506×, max = 1,660×) and paired normal (mean = 1,326×, min = 763×, max = 1,727×) samples. We then compared the allele frequencies of variants discovered during the original and/or subsequent deep sequencing (Fig. 4). To focus on high-confidence events likely to be of biological relevance, we removed silent variants, those in intronic, intergenic, or flanking regions, those in *IGH* (and, hence, likely arising due to somatic hypermutation), or those that were flagged as likely germline variants by at least one caller in at least one study. This resulted in 57 variants in the original sequencing study (mean depth = 92×) and 67 variants in the subsequent study (mean depth = 1,169×). Variant allele frequencies (VAFs) of mutations shared across the two studies were highly correlated (*R*^2^ = 0.80; *p* < 2.2e-16). As expected, the vast majority of variants unique to either study had low VAFs: one of the four variants unique to the original study had a VAF <10%, though all had an alternate allele count of three or fewer supporting reads, while 12 of the 14 variants unique to the subsequent study had a VAF <10%. Though relatively few new variants were discovered by the additional sequencing, these did include several annotated in COSMIC in genes *KRAS*, *HECW1*, and *ZFHX4*.

These results were recapitulated in a comparison of the variants discovered in 44 samples subjected to capture-based sequencing (mean depth of 1,562 capture-based variants = 123×) and previously to exome sequencing (mean depth of 3,563 exome variants = 136×; Supplementary Figure S7). As in our above comparison, the 1,450 mutations discovered in both studies had highly correlated VAFs (*R*^2^ = 0.85; *p* < 2.2e-16). Again, the majority of variants unique to one study had low VAF: 79 of 112 variants unique to the capture-based study (Supplementary Fig. S7C) and 2,066 of the 2,113 variants unique to the exome-based study (Supplementary Figure S7B) had VAFs <10%.

To further explore the effects of sequencing depth, we downsampled the sequencing reads from the 15 deeply-sequenced samples, called variants on the downsampled reads, and plotted the total number of variants following filtering (as above) in the downsampled and full data set (Supplementary Figure S8). As expected, the number of variants was correlated with sequencing depth (*R*^2^ = 0.52; *p* = 10^−3^). However, beyond ~25% of the final sequencing depth (or a mean depth of ~300×), the increase in number of discovered variants is marginal. Similar performance was observed at an even lower depth of 92× in the aforementioned case of the 15 original capture results later re-sequenced. Together, these results indicate that depths as low as 100× can capture the majority of variants of interest and that coverage beyond 300× will lead to sharply diminishing returns.

### Targeted capture sequencing facilitates integrative analysis across mutation types

**Fig. 5:**
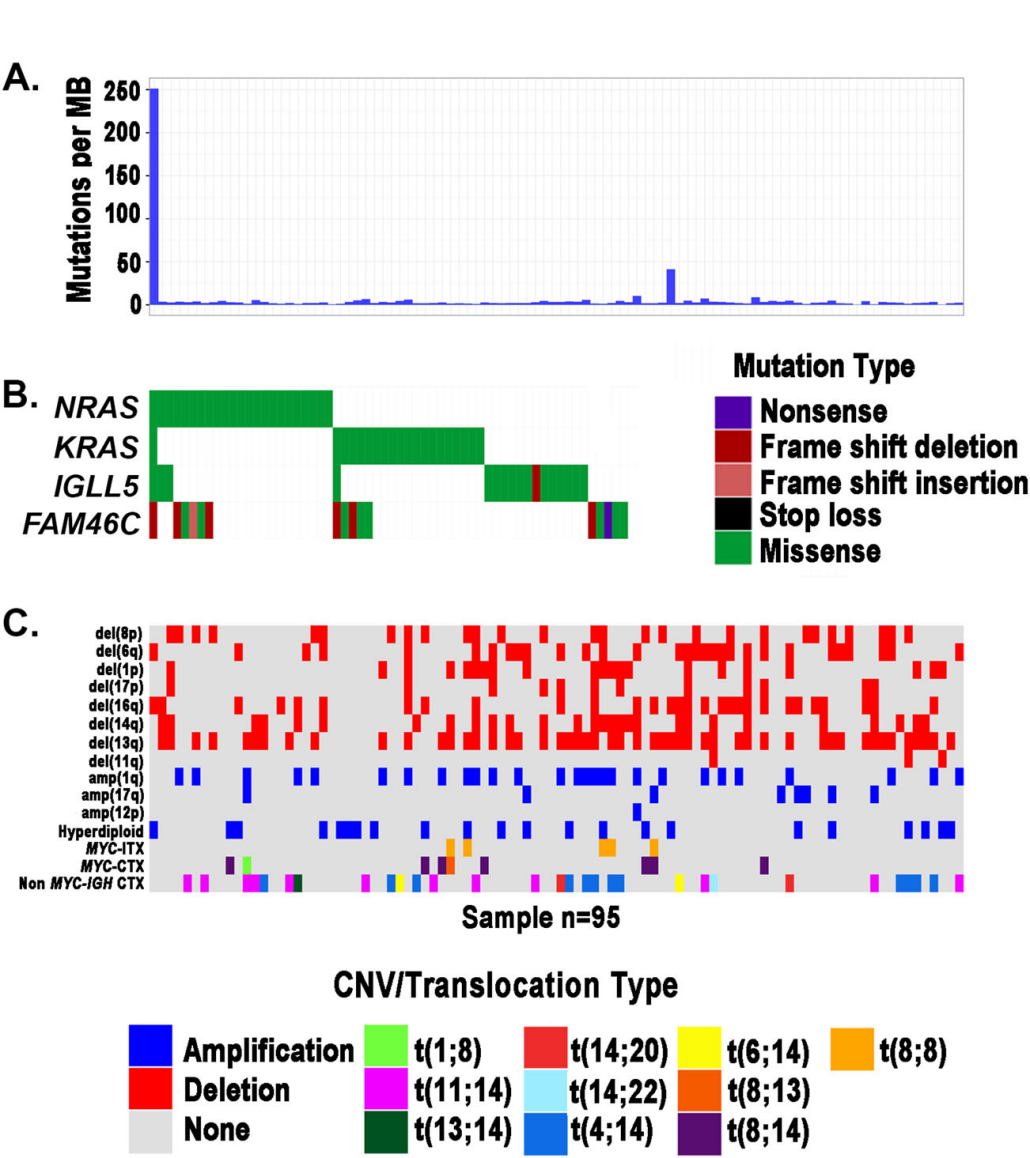
Targeted sequencing identifies CNVs, SNVs, and translocations. **a** Mutations per Mb, **b** SNVs, and **c** CNVs and translocations detected across 95 samples (columns). *MYC*-ITX: intra-chromosomal *MYC* translocations; *MYC*-CTX: inter-chromosomal *MYC* translocations; Non *MYC*-*IGH* CTX: inter-chromosomal *IGH* translocations, excluding those involving *MYC*

**Fig. 6:**
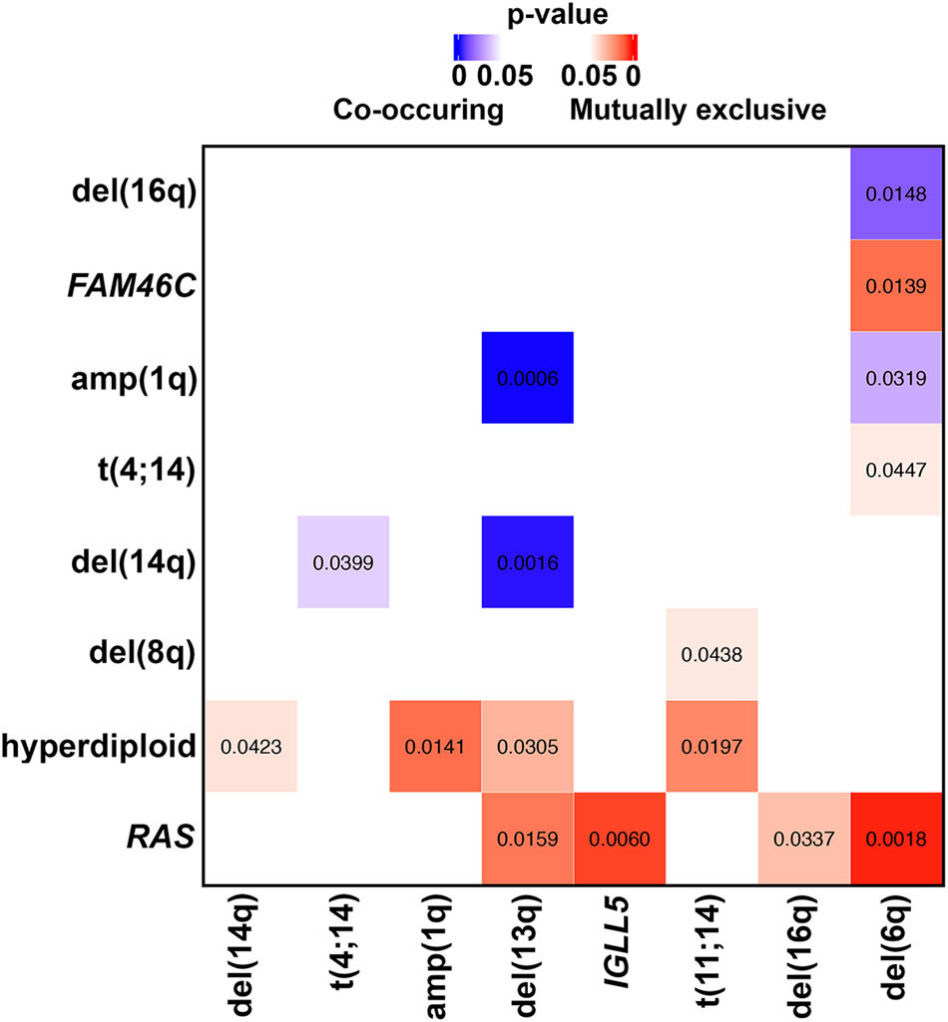
Targeted sequencing identifies co-occurrence and mutual exclusivity across mutation types. Co-occurring (blue) and mutually exclusive (red) mutations (*p* < 0.05). Numbers indicate *p*-values

**Fig. 7:**
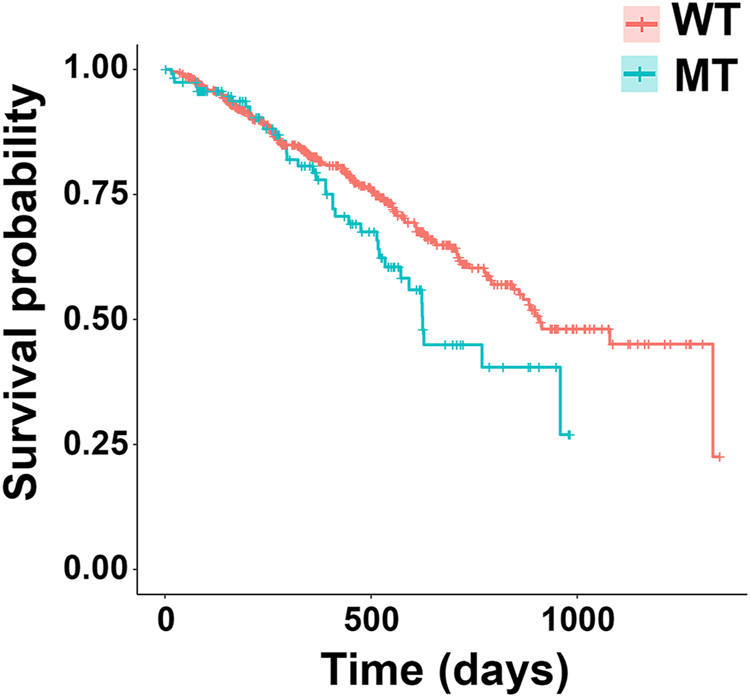
*IGLL5* mutations are associated with increased risk of disease progression. Kaplan-Meier curves of *IGLL5* mutant samples (with non-synonymous SNVs and/or indels) versus *IGLL5* WT samples

Integrated analysis of CNVs, SNVs, and translocations highlights patterns of mutual exclusivity and co-occurrence both within and across mutation types (Fig. 5). We tested for significance of these patterns after excluding the apparent hypermutator sample (leftmost column; Fig. 5a) to improve statistical power. This revealed mutation co-occurrence (blue; Fig. 6; Table S9) within CNVs [i.e., of del(6q) with del(16q) and amp(1q) and of del(13q) with amp(1q) and del(14q)] and involving CNVs and translocations [i.e., of del(14q) with t(4;14)]. As expected, we detected mutual exclusivity (red; Fig. 6; Table S9) between hyperdiploidy and t(11;14). We also detected cross-mutation type exclusivity between CNVs and SNVs [i.e., both *RAS* mutations (i.e., *KRAS* or *NRAS*) and *FAM46C* are mutually exclusive with del(6q)]. *IGLL5* was the third most frequently mutated gene in our data set (Fig. 5b; 18%), with (silent and non-synonymous) *IGLL5* mutations enriched for a c-AID signature (i.e., C to T/G mutation at WRCY motifs; *p* = 2.7 × 10^−6^; binomial test). Thirty-eight of the 40 detected *IGLL5* SNVs occurred in amino acid positions one to 100, outside of any annotated protein domains. The remaining two (K166N and K189R) occurred in the immunoglobulin C1-set domain. Mutations in *IGLL5* were mutually exclusive of *RAS* mutations (*p* = 0.006), with trends toward mutual exclusivity with *KRAS* (*p* = 0.054), *NRAS* (*p* = 0.111), and *FAM46C* (*p* = 0.113), independently (Figs 5 and 6). *IGLL5* mutations in diploid loci had a median VAF of 58% and a first quartile VAF of 39%, suggesting that the majority are likely clonal (Supplementary Figure S9). Finally, we found that *IGLL5* SNVs are associated with disease progression [Fig. 7; hazard ratio = 1.46 (95% confidence interval: 1.03–2.08); *p* = 0.03 (log-rank test)].

## Discussion

We developed a MM-specific, capture-based sequencing platform targeting 465 genes that detects CNVs, SNVs, and translocations. We used the platform to sequence 95 tumor/normal pairs and validated its ability to detect SNVs and translocations using exome sequencing and FISH data. After developing custom computational approaches for reducing false positives, we detected CNVs (Fig. 1) and known *IGH* translocations at expected frequencies (Fig. 2; Table 1) and discovered rare novel chromosomal translocations (Fig. 3). By deeply sequencing 15 pairs, we showed that there is a marginal increase in number of non-silent variants discovered with sequencing depth (Fig. 4). Hence, many scientific and some clinical goals may be achievable with the more modest sequencing depth (~100×) used for the complete cohort of 95 pairs.

We detected (intra- and inter-chromosomal) *MYC* translocations at a frequency of 13% [95% CI (7%-20%); 12 of 95; Fig. 2; Tables S5 and S7]. This is generally consistent with prior studies, though a considerable range of frequencies has been published. Such discrepancies are likely due in part to differences in sample size, disease stage, assay, and assay optimization/tuning. An early study using FISH reported complex *MYC* abnormalities in 50% of primary tumors—however, the analyzed samples were few [7 of 14; 95% CI (26%-74%)] and advanced (stage III)^32^. *MYC* translocation frequencies more similar to those we observed have been detected in newly diagnosed MM patients using both FISH—15% [79 of 529; 95% CI (12%-18%)]^33^ and 23% [62 of 274; 95% CI (18%-28%)]^34,35^—and a capture-based next-generation sequencing approach similar to that presented here—20% [21 of 104; 95% CI (13%-28%)]^14^.

Our data revealed that *IGLL5* is mutated via both point substitutions and translocations in MM (Figs 2, 3 and 5). The SNVs within the locus likely result from somatic hypermutation of the immunoglobulin lambda light chain locus, which spans *IGLL5*. Indeed, we saw an enrichment for AID-induced mutations within *IGLL5*, as has been previously observed in chronic lymphocytic leukemia (CLL), where the gene is frequently mutated^36^. We found that non-synonymous *IGLL5* mutations are both mutually exclusive with *RAS* mutations (Fig. 6) and associated with disease progression (Fig. 7) and, with a median VAF of 58%, are likely present in the founding clone. Together, these findings suggest that *IGLL5* mutation may contribute to myeloma pathogenesis, but without additional data their functional significance remains unknown. The fact that mutations and translocations do not recurrently affect specific residues in the IGLL5 protein supports a loss of function model, but an alternative hypothesis is that *IGLL5* mutations are simply a biomarker for high-risk disease.

We observed that *IGLL5* was translocated to the *IGH* locus in two patient samples (Figs. 2 and 3). Translocations between *IGH* and either of the two light chain loci have been reported in B cells and their associated disorders: *IGL*/*IGH* translocations have been reported in non-Hodgkin lymphoma^37^ and in activated B cells^38^ where it was suggested that the translocations resulted from deficiencies in non-homologous end-joining that induced both V(D)J-recombination-associated breaks at the *IGL* locus and class switch recombination-associated *IGH* breaks. The authors speculated that, since the translocations conferred no obvious selective advantage, these translocations simply reflected the mechanistic opportunity presented by two frequently-broken and spatially-proximal loci^38^. *IGH* translocations partnered instead with *IGK* have been detected in B-cell lymphomas^39^ and in a patient-derived B lymphoblastoid cell line^40^ with the latter plausibly attributable to the patient’s prior, long-term treatment with a DNA-damaging alkylating agent. The possibility remains that the translocations we detected play a functional role by co-opting a large super-enhancer downstream of *IGLL5*, which was found to be active within the MM1.S MM cell line^41^. Its position between *IGLL5* and *BCR* place it on the der(14) chromosome of the t(14;22) translocation we validated. Hence, this super-enhancer may amplify the effect of the *IGH* enhancers in upregulating the expression of nearby genes translocated to chromosome 14. Indeed, we observed upregulation of one such gene, *DERL3*, across MM tumors relative to other cancer types, generally (Supplementary Figure S5), and in the sample with the second putative t(14;22) translocation, specifically (Fig. 3). Overexpression of *DERL3* could play a role in MM by increasing endoplasmic reticulum (ER)-associated degradation within the proteasome. *DERL3* forms an export channel in the ER through which misfolded proteins fated for degradation reach the proteasome^42^. The proteasome inhibitor bortezomib is highly efficacious in MM cells. Hence, were it to instead promote, rather than inhibit, activity of the proteasome, *DERL3* overexpression might confer a selective advantage to MM cells. Future experiments are required to decipher the importance of *IGLL5* translocations and the significance of *DERL3* overexpression.

In addition to detecting known mutations, the platform was designed to enable discovery: (1) We queried 465 genes, a much larger set than assayed by previous targeted platforms^6,11,16^, and (2) we tiled across the entire V, D, and J regions, as opposed to restricting probes to annotated segments within these regions^15^ in an attempt to detect translocations involving inter-segment regions of the locus. Additional experience will be necessary to evaluate these design decisions. For example, lack of inferred translocations involving the J region may suggest that probes used to tile across this ~700 Kb region are better invested elsewhere. In particular, future versions of the platform should provide currently lacking coverage of chromosome arm Yq, though the tandem-rich arms of acrocentric chromosomes (13p, 14p, 15p, 22p, and Yp) will remain difficult to capture. Additionally, targeting probes to common SNPs would enable detection of allele-specific ploidy at a given locus^43^, which would facilitate SNV-based inference of clonal evolution^44^.

Mutation^4^ and expression^45^ data, coupled with ISS stage, have been shown to improve prognostic accuracy over ISS stage alone. Indeed, two recent crowd-sourcing competitions [a DREAM challenge (https://www.synapse.org/#!Synapse:syn6187098/wiki/401884; refs^46,47^ and a Topcoder challenge (http://crowdsourcing.topcoder.com/myeloma_predictor)] aim to improve MM patient stratification using genomic, transcriptomic/expression, cytogenetic, and clinical features. The ease of employing a single platform to assay CNVs, SNVs, and translocations would facilitate these and related efforts. Significantly, the same probes could be used to perform targeted RNA sequencing, which would also furnish these studies with comparative gene expression across samples.

## Electronic supplementary material

Supplemental Tables(XLS 13041 kb)

Supplemental Methods(PDF 158 kb)

Supplemental Figures(TIF 7876 kb)
