# Supplementary material for: A multiple myeloma-specific capture sequencing platform discovers novel translocations and frequent, risk-associated point mutations in IGLL5
Source: Blood Cancer J. 2018 Mar 21;8(3):35. doi: 10.1038/s41408-018-0062-y (PMC5862875; doi:10.1038/s41408-018-0062-y)
Supplement: Supplementary file 2 — Supplemental Methods(PDF 158 kb) [file 41408_2018_62_MOESM2_ESM.pdf]

SUPPLEMENTAL INFORMATION: DISCUSSION, METHODS, FIGURES, FIGURE LEGENDS, AND TABLES

SUPPLEMENTAL DISCUSSION

We designed probes targeting the exonic regions of canonical *IGH* translocation partners but did not design probes within the intronic or flanking intergenic regions of these genes where translocations often occur. Rather, *IGH* (and *MYC*) probes acted as bait, enriching for molecules that juxtapose a fragment of the *IGH* (or *MYC*) locus (complementary to the probe) and the partner gene. Paired-end sequencing then detects the partner gene when: (1) a chimeric (or split) read spans the breakpoint, thus revealing it at nucleotide resolution and/or (2) each of the two mates of a discordant paired-end read align to one of the partners, thus bracketing the breakpoint and defining a region over which it occurred. We hypothesized that endonucleolytic cleavage of free DNA ends prior to fusion with a partner chromosome could result in translocation breakpoints upstream of the double-stranded break. Hence, even if double-stranded breaks occurred at the boundaries of or within the *IGHV*, *IGHD*, *IGHJ* genes, or the switch regions, the translocation breakpoint may not respect these boundaries. As such, we allowed probes to lie entirely outside, entirely inside, or partially outside/inside these genomic elements.

SUPPLEMENTAL METHODS

Custom capture sequencing platform design

We designed a custom Nimblegen probe set (Roche), targeting 3.3Mb of space that includes 465 genes and the *IGH* region. ~1.3Mb of the capture space spans the *IGH* locus and ~160Kb spans the *MYC* locus. Probes were designed from ~50Kb upstream to ~50Kb downstream of the *IGH* locus (chr14:105982580-107289508; hg19 coordinates). 85% of this region was nominally covered by probes, though additional coverage was provided by the wingspan of

these probes. Probes were also designed across exonic and intronic regions of the *MYC* locus spanning ~50Kb upstream to ~100Kb downstream (chr8:128697680-128853674; hg19 coordinates). 70% of this region was covered by probes. All chromosomes arms have coverage except 13p, 14p, 15p, 22p, Yp, and Yq. As noted in the Discussion, all of these except Yq are tandem-rich arms of acrocentric chromosomes.

Capture sequencing of 95 tumor/normal pairs

Automated dual indexed libraries were constructed with 100-250ng of genomic DNA utilizing the KAPA HTP Library Kit (KAPA Biosystems) on the SciClone NGS instrument (Perkin Elmer) targeting 250bp inserts. 96 libraries were pooled pre-capture generating a 5µg library pool. Library pools were hybridized with the Nimblegen probe set. The concentration of each captured library pool was accurately determined through qPCR according to the manufacturer's protocol (KAPA Biosystems) to produce cluster counts appropriate for the Illumina HiSeq2000 platform. Two lanes of 2x100 sequence data were generated per library pool. One of the original 96 samples was subsequently excluded because of low coverage.

Deep capture sequencing of 15 tumor/normal pairs

Fifteen tumor/normal pairs (a subset of the original 96 samples sequenced) were subjected to additional sequencing in three batches. Six pairs were subjected to two rounds of sequencing and the remaining nine pairs to a single round. Both rounds of sequencing for the first six pairs utilized existing libraries created during the initial sequencing of the 96 tumor/normal pairs. In the first round of sequencing, one library pool was created for capture (total library yield into the hybridization was 2.5µg and included all 12 libraries) and was sequenced on one lane of HiSeq2500 (2x125 reads). Similarly, for the second round, one library pool was created for capture (total library yield into the hybridization was 4.8µg and included all 12 libraries) and was

sequenced on the Rapid Run mode on the HiSeq2500 (two lanes on one flow cell generating 2x100 reads).

Deep sequencing of the final nine sample pairs was performed by first constructing automated dual indexed libraries with 250ng of genomic DNA utilizing the KAPA HTP Library Kit (KAPA Biosystems) on the SciClone NGS instrument (Perkin Elmer) targeting 250bp inserts. Four independent 3μg library pools were created from nine cases including both tumor/normal libraries. Each library pool was hybridized with the custom Nimblegen probe set. The concentration of each captured library pool was accurately determined through qPCR according to the manufacturer's protocol (KAPA Biosystems) to produce cluster counts appropriate for the Illumina HiSeq2500 1T platform (2x125 reads).

Sequencing coverage calculation

Average depth and on-target efficiency were calculated using the Genome Modeling System's[1] utilities for measuring depth and alignment coverage. These tools rely on the RefCov software suite (http://gmt.genome.wustl.edu/gmt-refcov), which provides a number of methods for analyzing nucleotide sequence coverage. RefCov calculates summary and per-base position coverage statistics relative to a reference genome based on an input alignment BAM file.

Reported mean, minimum, and maximum coverage statistics are based on on-target bases—i.e., bases aligned within the coordinates of the designed probes, as specified by the BED file (**Table S1**). Hence, no bases within the wingspan of the probes were considered on-target. Per-base coverage was then calculated for each base in the target space. A sample's mean coverage was then calculated as the mean per-base coverage across all bases having at least 1X coverage. The reported mean, minimum, and maximum coverages are then the mean, minimum, and maximum across all samples of the per-sample mean coverages. In addition to

total aligned on-target bases, **Figures S1** and **S2** show total duplicate bases (i.e., total number of on-target bases occurring in duplicate reads), total aligned off-target bases (which includes both unique and duplicate reads), and total unaligned bases. Finally, percent on target efficiency at a specified depth (30X in **Figures S1** and **S2**) was calculated by first summing the total coverage at on-target bases that met or exceed the specified depth and then by dividing this sum by the total number of bases sequenced.

Capture sequencing-based copy number detection

Copy number variants (CNVs) were called using CopyCAT2 (https://github.com/abelhj/cc2/; Ref. 2) parameterized to detect copy number alterations exceeding the level of noise estimated from diploid regions using a gaussian mixture model (https://github.com/genome/bmm). CopyCAT2 was specifically developed to detect CNVs from capture sequencing data. CNVs were called if the (nominal) binomial *p*-value output by CopyCAT2 (p.cov.np) was less than 0.05, which is computed based on the number of capture probes having a tumor/normal depth log ratio outside some upper or lower limit. These upper and lower limits were defined as the mean plus or minus, respectively, two times the standard deviation of the distribution of log ratios from a "typical" diploid region. Chromosome 2 was generally used as the reference diploid region, as it is infrequently altered in multiple myeloma. In several instances, however, chromosome 2 proved too noisy and a different chromosome was used as the diploid region, namely: chromosome 10 for samples H_QD-WAPAT023-V0DHO9, H_QD-WAPAT025-V0DHOD, H_QD-WAPAT030-V0DHON, and H_QD-WAPAT032-V0DHOR and chromosome 17 for samples H_QD-WAPAT082-V0DHRJ, H_QD-WAPAT052-V0DHPV, H_QD-WAPAT056-V0DHQ3, and H_QD-WAPAT014-V0DHNR. Additionally, two samples were excluded from copy number analysis: H_QD-WUPAT001-V0DHMT had low coverage and H_QD-WUPAT002-V0DHMW showed poor correlation between tumor and normal.

To prevent the upper and lower limits from being overly sensitive to potential focal and/or arm-level CNVs within the supposedly diploid region, a gaussian mixture model (i.e., sum of gaussian distributions) was fit to the tumor/normal ratios (not their log-transformed values) within the region (**Figure S3**). Since the bulk of the region was assumed diploid, the gaussian making the largest contribution to the mixture (i.e., with the largest weight and hence representing the most probes) was taken as a model of the unaltered subregions. The gaussian mixture model was fit using a variational Bayesian approach implemented in the R bmm package (https://github.com/genome/bmm) and used previously as the backend of SciClone—a method for inferring clonal evolution from sequencing data.[3] The mixture was fit with two gaussian components by: invoking init.gaussian.bmm.hyperparameters with N.c = 2, passing the resulting initialized hyperparameters to init.gaussian.bmm.parameters, and finally passing the parameters resulting from that call, along with the hyperparameters, to gaussian.bmm. gaussian.bmm was also passed parameters convergence.threshold = 10^{-4}, max.iterations = 10000, and pi.threshold=10^{-2}. The data passed to these functions was output by an initial call of CopyCAT2. CopyCAT2 was invoked independently for each tumor sample, with the corresponding normal sample used as the (single) control sample, and with parameters coverage.min.ratio = 0.125, coverage.max.ratio = 8, min.num.normals = 1, min.norma.corr = 0.5, segalpha = 0.05, and vafs_normalize = FALSE. Additionally, the BED file describing the custom capture probes (**Table S1**) was passed as the target.bedfile parameter after first excluding *IGH* coordinates. This effectively prevents CopyCAT2 from attempting to call CNVs within the *IGH* locus. This BED file was used to calculate coverage using bedtools coverage, which was subsequently passed to CopyCAT2.

Following fitting of the gaussian mixture, CopyCAT2 was invoked a second and final time to detect CNVs based on the margins established by the fit. Parameters were as specified above in the initial run, except with coverage.min.ratio and coverage.max.ratio set to the mean (which

is one, since CopyCAT2 mean centers the data) plus or minus twice the standard deviation of gaussian model of the unaltered regions.

CNVs output by CopyCAT2 were annotated to indicate whether they were amplifications (CopyCAT2 finalcn field > 2) or deletions (finalcn < 2), whether they were focal or arm-level, whether they participated in a hyperdiploid event, and, for focal CNVs, what genes they encompassed. A CNV was annotated as belonging to a chromosome arm if at least one breakpoint was within that arm; it was labeled as "arm"-level if its length was at least half the length of the *targeted* region of the arm and "focal" otherwise (**Table S4**). An event that involved both arms of the chromosome was annotated for both the p- and q-arms, with a separate entry in the table (**Table S4**) for each. A sample was considered hyperdiploid if it had amplifications of at least five of the chromosomes 3, 5, 7, 9, 11, 15, 19, and 21 (i.e., both p- and q-arms, except for chromosome 15, since 15p was not targeted). Focal CNVs were annotated with (hg19) genes they encompassed using findOverlaps from the GenomicRanges R package.

Figure 1 shows hyperdiploid and focal copy number events detected by CopyCat2 (blue; $p<0.05$) from $\log_2$ ratios of tumor to paired normal sequencing depth across chromosomes. (Clonal) single-copy gains occur at a $\log_2$ ratio of $\log_2(3/2) \sim 0.58$, whereas (clonal) heterozygous/single-copy losses occur at a $\log_2$ ratio of $\log_2(1/2) = -1$. Homozygous losses occur, in principle, at $\log_2(0/2)$ or negative infinity. The finite negative ratio of the homozygous focal loss (**Figure 1B**) may indicate that it is subclonal and/or reflect a small number of spurious alignments to this region of the genome.

Capture sequencing-based translocation detection

Translocations were detected using LUMPY (https://github.com/arq5x/lumpy-sv; Ref. 4), with results filtered by a machine learning approach optimized to achieve high precision relative to

available FISH results. First, FASTQ files were aligned against the human genome (hg19) using the aln command of SpeedSeq[5] (v0.0.1) and parameters "-t 4 -o prefix," which resulted in three BAM files: prefix.bam containing all alignments, prefix.splitters.bam containing all split reads, and prefix.discordants.bam containing discordant read pairs. The empirical insert size distribution was calculated for each alignment BAM file using the pairend_distro.py utility distributed with LUMPY. Specifically, samtools[6] was used to output the entries of the prefix.bam file, with the first 10,000 entries skipped and the remainder piped to pairend_distro.py with parameters "-X 4 -N 10000," with results output to prefix.hist. The mean m and standard deviation sd of the insert size for prefix.bam were parsed from prefix.hist and used to define back_dist = $m + 3 * sd$. LUMPY (v0.2.13) was then invoked independently for each patient, with paired-end "pe" and split read "sr" arguments for each discordant and split-read BAM file (for both tumor and normal samples), respectively, associated with that patient. Specifically, each prefix.bam file associated with the patient resulted in a set of arguments: "-pe id:<sample_name>,bam_file:prefix.discordants.bam,histo_file:prefix.hist,mean:m,stdev:sd,read_length:<read_length>,min_non_overlap:<read_length>,discordant_z:5,back_distance:back_dist,weight:1,min_mapping_threshold:<threshold>" and "-sr id:<sample_name>,bam_file:prefix.splitters.bam, back_distance:back_dist,weight:1,min_mapping_threshold:<threshold>," where <sample_name> was the name of the sample, <threshold> was 50, and <read_length> was the mode of the lengths of the first 110,000 reads (as determined by outputting the first 110,000 reads of prefix.bam using samtools), which was 100. Translocations were annotated with the nearest cancer-associated gene (as cataloged in the cancer gene census[7]) within 1Mb of either breakpoint.

Putative translocations involving *IGH* (defined as those with a partner within the region chr14:105982614-107338051) or *MYC* (chr8:128697680-128853674) were parsed out of the

LUMPY VCF (variant call format) output using a custom script. In addition to the indicated coordinates, putative translocations were required to include a VCF MATEID field and SVTYPE=BND (indicating a complex rearrangement with two breakends).

Each putative inter-chromosomal *IGH* translocation was further filtered using a support vector machine (SVM) trained on available FISH data and using as input the number of split reads (indicated by the SR=<num_reads> field of the LUMPY VCF output file) and the number of paired-end reads (indicated by PE=<num_reads>) supporting the translocation. The SVM was trained to perform binary discrimination between putative *IGH* translocation calls that were and were not validated by FISH. Only those LUMPY inter-chromosomal *IGH* translocation calls involving (non-*MYC*) canonical partners were used during the SVM training and test phases—i.e., those with one breakpoint on chromosome 14 within the region spanning from 1MB up- to 1MB down-stream of *IGH* (chr14:105982614-107338051) and with a second breakpoint on one of the canonical *IGH* partner chromosomes, spanning from 1MB up- to 1MB down-stream of genes previously implicated in *IGH* translocations[8] on chromosomes 4 (near genes *FGFR3*, *LETM1*, or *WHSC1*), 6 (*CCND3* or *UBR2*), 11 (*PPP6R3*, *TPCN2*, *MYEOV*, or *CCND1*), 16 (*WWOX* or *MAF*), or 20 (*DHX36*, *LOC339568*, or *MAFB*) (**Table S5**). A LUMPY call of a canonical *IGH* translocation was considered validated by FISH if the corresponding partner was detected in a tumor sample at the Mayo Clinic and/or the sample collection site. In no case did FISH performed at the Mayo Clinic and the sample collection site detect different *IGH* translocations. A LUMPY call of an *IGH* translocation was not considered validated by FISH if it was called within one of the paired normal samples (which were assumed not to harbor translocations) or if it disagreed with the translocation inferred by either site. The requirement that LUMPY and FISH not disagree effectively implies the assumption that a patient sample not harbor multiple *IGH* translocations—with the exception of a secondary t(8;14) translocation, which were not considered during SVM training.

Tuning of a linear SVM was performed with five-fold cross-validation in Python using the scikit-learn library. Specifically, LUMPY calls of canonical *IGH* translocations in tumor samples subjected to FISH assay or in the corresponding normal samples were partitioned into equally-sized training and test sets, stratified by whether they were or were not validated by FISH using train_test_split with the stratify parameter. At most one LUMPY call involving each partner chromosome was considered during the training and test phase—if LUMPY inferred multiple calls involving the same partner, only that call with the largest total evidence (number of supporting split reads plus number of supporting paired reads) was considered. LUMPY calls in normal samples were, by assumption, not considered validated by FISH. The C parameter of the linear SVM was tuned on the training data via a grid search over the values C = $\{10^{-4}, 10^{-3}, 10^{-2}, 10^{-1}, 1, 10, 10^{2}, 10^{3}, 10^{4}\}$ and using five-fold cross-validation with GridSearchCV(SVM(kernel='linear', C=1), [{ 'C' : 10**x for x in range(-4,4)]}], cv=5, scoring='precision_micro'). The best fit was obtained with C = 0.1, which was subsequently applied to the held-out test samples to evaluate precision and recall and to call *IGH* translocations across all samples, including those involving *MYC* and non-canonical partners. t(8;14) translocations were those LUMPY calls that passed the SVM filter and that had a breakpoint within 1MB up- or down-stream of *MYC*.

FISH results for *MYC* translocations were not available for filtering LUMPY results. Hence, we manually defined a decision boundary in the space of number of supporting split reads and paired-end reads to separate those LUMPY calls that were likely to be false positives (in particular, those that were detected in normal samples) from those more likely to be true positives. To do so, independently for intra- and inter-chromosomal *MYC* translocations, we defined the separating hyperplane such that all translocations inferred in normal samples were assigned to the likely false positive class. Specifically, we manually selected translocations

based on their numbers of supporting reads that should be used to define the boundary (i.e., a subset of which would be selected as support vectors to define the hyperplane). This was accomplished by defining an SVM via SVM(kernel='linear', C=1) and then by invoking its fit method with a sample_weight argument that assigned a non-zero weight (10) to the manually-selected translocations and zero weight to all others. In the case of intrachromosomal translocations, the manually selected false positive translocation was at (number of split reads = 2, number of paired-end reads = 2) and the true positive translocations were at (7, 6) and (0, 7). Note that the plotted numbers of supporting reads have been jittered so that overlapping translocations are visible. In the case of interchromosomal translocations, the manually selected false positive translocation was at (8, 8) and the true positive translocation was at (28, 39). This approach ensured that we could identify putative (but presumably false positive) *MYC* translocations in normal samples with 100% precision based on number of supporting split and paired-end reads. We subsequently filtered any *MYC* translocations in *tumor* samples that were assigned by the SVM to the same class as the normal sample translocations.

Mapping of *IGH* constant, switch, and enhancer regions

The following genes were searched on Ensembl GRCh37: *IGHA2*, *IGHE*, *IGHG4*, *IGHGP*, *IGHA1*, *IGHEP1*, *IGHG1*, *IGHG3*, *IGHD*, *IGHM* and the "Location" of each gene served as the *IGH* constant regions. We identified switch regions as those regions enriched for repeats downstream of the constant regions. To do so, we entered the coordinates of the constant regions above into the UCSC Genome Browser (http://genome.ucsc.edu/cgi-bin/hgGateway) with the following parameters: a. Group: Mammal b. Genome: Human and c. Assembly: Feb. 2009 (GRCh37hg19). Dropdown controls were used to hide everything except:

a. Mapping and Sequencing > Base position > Full

b. Genes and Gene Prediction > USCS Genes > Full

c. Genes and Gene Predictions > Ensembl Genes > Full

d. Genes and Gene Predictions > Vega Genes > Full

e. Repeats > Simple Repeats > Full

This displays simple tandem repeats located by Tandem Repeats Finder. Repeat regions located downstream of the constant region were treated as the switch regions for the adjacent constant region. Some of the switch regions were separated into non-contiguous tandem repeats with repeating sequences, so the whole region spanning these repeats was treated as the switch region. To confirm the validity of this approach, we used BLAT to map the known[9] sequence (GenBank: X54713.1) for the mu switch region to the hg19 coordinates chr14:106322327-106326797. This corresponds closely to those we inferred using the above tandem repeat-based method (chr14:106323230-106326599).

The 3' enhancer region coordinates were determined by using BLAT to map the reported[10] enhancer elements. The range of the four sequences downstream of *IGHA1* (GenBank: AF013718.1, AF013722.1, AF013722.1, AF013725.1) was determined to be chr14:106152458-106167601 (hg19 coordinates) and used to define the "E3A1" enhancer. Similarly, the three sequences downstream of *IGHA2* (AF013719.1, AF013724.3, AF013726.1) mapped to a range chr14:106032614-106048676 that we used to define the "E3A2" enhancer. The mu enhancer region coordinates were determined by using BLAT to map the sequence TTTTTTAATTAATTGAGCGAAGCTGGAAGCAGATGATGAATTAGAGTCAAGATGGCTGCATG GGGGTCTCCGGCACCCACAGCAGGTGGCAGGAAGCAGGTCACCGCGAGAGTCTATTTTA GGAAGCAAAAAAACACAATTGGTAAATTTATCACTTCTGGTTGTGAAGAGGTGGTTTTGCCC AGCCCAGATCTGAAAGTGCTCTACTGAGCAAAACAACACCTGGACAATTTGCGTTTCTAAA ATAAGGCGAGGCTGACCGAAACTGAAAAGGCTTTTTTTAACTATCTGAATTTCATTTCCAAT CTTAGCTTA reported in Fig. 5 of Ref 11.

Validation of novel t(14,22) translocation

Polymerase chain reaction (PCR) was performed on two nanograms of genomic DNA isolated from CD138^{+} selected bone marrow (tumor) biopsy and peripheral blood leukocytes (germline) from the patient that was called positive for the t(14;22) translocation by capture sequencing. Primers were designed to span the translocation breakpoints on the derivative chromosomes based on the base-pair resolution reads from capture sequencing. Reactions were run using GoTaqGreen Master Mix (Promega) per manufacturer's instructions with oligos designed to detect derivative chromosome 14 [Forward: 5'ACCACTAACAGGGGACATGC and Reverse: 5'TTTGATTATTCCCCCAACCA] and derivative chromsome 22 [Forward: 5'ACAAGCCAGAGGAGTGAGGA and Reverse: 5'CTCTGAAGACCAGGCTCACC]. PCR products were separated with DNA electrophoresis; products specific to tumor samples and of the expected size were cut out and DNA was isolated using the Zymoclean Gel DNA Recovery Kit (Genesee Scientific) per manufacturer's instructions and sequenced using the same primers as the PCR reactions. The sequences were mapped to the human genome (GRCh37 assembly) using BLAT (UCSC genome browser) and alignments with genomic locations matching breakpoints obtained from capture sequencing were identified to confirm the presence of t(14;22) translocation in the gDNA.

The quality of DNA was checked by detecting the presence of chromosomes 14 and 22 wild-type for the translocation in the same DNA samples mentioned above. For the translocation, two breakpoints were present on each chromosome and the region between the breakpoints was deleted from the translocated chromosomes. Oligos were designed that spanned the deleted section of chromosomes 14 [Forward: GGGCTGTGTCTCTGTGGTAT and Reverse:GTGGAATGTGTGTGAGCTGG] and 22 [Forward: ATAGGGTCCGTGCACCATTC and Reverse: ATGCTGAGCTAACCACCCTT], and PCR products were run on an agarose gel.

<u>Validation of novel t(13;14) translocation</u>

Polymerase chain reaction (PCR) was performed on two nanograms of genomic DNA isolated from CD138+ selected bone marrow (tumor) biopsy and peripheral blood leukocytes (germline) from the patient that was called positive for the t(13;14) translocation by capture sequencing. Primers were designed to span the translocation breakpoints on the derivative chromosomes based on the base-pair resolution reads from capture sequencing. Reactions were run using GoTaqGreen Master Mix (Promega) per manufacturer's instructions with oligos designed to detect derivative chromosome 13 [Forward: 5'AATCTTTCTGTTCTGTTGGCATT and Reverse: 5'AATCTTTCTGTTCTGTTGGCATT]. PCR products were separated with DNA electrophoresis; products specific to tumor samples and of the expected size were cut out and DNA was isolated using the Zymoclean Gel DNA Recovery Kit (Genesee Scientific) per manufacturer's instructions and sequenced using the same primers as the PCR reactions. The sequences were mapped to the human genome (GRCh37 assembly) using BLAT (UCSC genome browser) and alignments with genomic locations matching breakpoints obtained from capture sequencing were identified to confirm the presence of t(13;14) translocation in the gDNA.

Somatic single nucleotide variant detection

Reads were aligned against human reference genome GRCh37-lite using BWA.[12] The SNV-calling pipeline used a combination of samtools,[6] SomaticSniper v. 1.0.4,[13] MuTect 1.1.4,[14] Strelka v. 1.0.11,[15] and VarScan version 2.3.6 (Ref. 16). To obtain a final set of calls, the somatic variation detection pipeline executes a series of union and intersection mergers to integrate the results of these tools.

First, SNVs are called using SAMtools version r982 (parameters: mpileup -BuDS) filtered by snp-filter version v1 and false-positive-filter v1 (parameters: –max-mm-qualsum-diff 100 --bam-readcount-version 0.4 --bam-readcount-min-base-quality 15) and intersected with Somatic Sniper version 1.0.4 (parameters: -F vcf -G -L -q 1 -Q 15) filtered by false-positive v1

(parameters: --bam-readcount-version 0.4 --bam-readcount-min-base-quality 15) then somatic-score-mapping-quality v1 (parameters: --min-mapping-quality 40 --min-somatic-score 40). A union join of these results is then performed with the output of the following 3 callers: (1) VarScan 2.3.6 (parameters: --nobaq --version r982) filtered by varscan-high-confidence v1 then false-positive v1 (parameters: --bam-readcount-version 0.4 --bam-readcount-min-base-quality 15); (2) Strelka version 1.0.11 (parameters: isSkipDepthFilters = 1); (3) MuTect 1.1.4 (parameters: --number-of-chunks 50;--cosmic-vcf b37_cosmic_v54_120711.vcf --dbsnp-vcf snvs.hq.vcf). The b37_cosmic_v54_120711.vcf represents the 1000-genomes format of the variants contained within COSMIC, while snvs.hq.vcf contains known the dbSNP variants from human build 142.

In addition to producing the standard position and base pair change of a variant, the somatic variation pipeline produces both a classification for mutation type (e.g., silent, missense, nonsense) as well the reference and alternate read counts and variant allele frequencies for both the tumor and matched normal samples. Together, this information provided a means of stratifying variants by relative importance and of assessing the sensitivity of the custom capture platform to detect low-frequency mutations.

Comparison between initial capture and subsequent deep sequencing

We explored the sensitivity afforded by increased sequencing depth by performing additional sequencing of 15 tumor (mean depth=1,259X, min=506X, max=1,660X) and paired normal (mean=1,326X, min=763X, max=1,727X) samples. We then performed a comparison of variants discovered by the initial and deeper sequencing. Both data sets were processed using the same pipeline parameters as detailed above. The final SNV variant calls were then compared to look for commonalities as well as those unique to each set.

Several filtering steps were carried out prior to the comparison of variants. This served to both highlight the genes of interest, as well as to account for the additional information provided by deeper sequencing. This additional information influences SNV results both by revealing rare low-frequency variants as well as by identifying potential contamination in the original lower-coverage results. For instance, a SNV with low variant allele coverage in the initial sequencing may be called if reference coverage is also low, so that the resulting VAF is appreciable and exceeds the caller's threshold. However, if deeper sequencing leads to additional coverage of that reference allele, without a corresponding increase in the variant allele, the resulting VAF may fall below a caller-required threshold and be filtered. This situation may indicate the variant reads are artifacts.

The following variants were removed prior to comparison between initial and subsequent deep sequencing:

1. Those annotated as intronic, intergenic, silent, or 5' flanking (to focus the comparison on those variants most likely of biological importance).
2. Those occurring in the *IGH* region (as these are likely caused by physiological somatic hypermutation and are not of biological significance).
3. Those rejected by a caller as likely germline in either data set.

<u>Sequencing downsampling</u>

We explored the effect of varying read depth on variant discovery by downsampling the deep sequencing data sets. Comparisons were performed at 25%, 50%, and 75% of the total coverage on the set of 15 samples for which additional sequencing was performed. The original BAM files containing all instrument data were first query-sorted using the SortSam utility from the Picard 1.138 toolkit (parameters: SORT_ORDER=queryname VALIDATION_STRINGENCY=LENIENT). To recreate the effects of lower coverage, the query-

sorted instrument data were then randomly down-sampled without replacement using the bam-sample tool from the fastq-tools package (version 0.8). Five repetitions of subsampling were performed on the 15 samples at all three levels of lower coverage. The reduced data sets were then imported into the standard somatic variation pipeline (above) to maintain consistency with the samples having full coverage. SNV calls from the downsampled results were excluded from the comparison as above or if they were annotated as RNA mutations. After filtering, the results were compared to the full complement of calls from the 100% coverage data set. The number of variants that overlapped the full-coverage variants exactly in position and nucleotide change are indicated on the y axis of **Figure S10**.

Comparison between exome and capture sequencing

In order to establish performance against an existing platform, we compared the results of capture sequencing to those previously obtained via exome sequencing (dbGaP Study Accession: phs000348.v2.p1). We downloaded alignment BAM files from dbGaP, converted them to FASTQ files, and reprocessed the unaligned reads using the same alignment and variant discovery pipeline as used for the capture sequencing data (above). This ensured that discrepancies reported between the two studies were not an artifact of different bioinformatic pipelines, but rather reflected differences in the sequencing platforms employed. Though 79 pairs overlapped between the two studies, we were only able to reprocess the data from 44 pairs, which are reported here.

To address the issue of the capture platform's much more restricted coverage, the comparison was limited to only those coordinates nominally targeted by the probes (**Table S1**). To further equalize the comparison, we extended the specification to include only those regions in the exome and capture that possessed a baseline level of 10X coverage in at least 50% of the samples. Namely, a position was required to have at least a minimum of ten reads supporting it

in both the normal as well as the tumor samples, in at least half of the capture and half of the exome results. This provided a set of positions between both platforms where affinity is consistent. The bedtools (version 2.17.0) utility multicov was used to extract the read counts from both tumor and normal samples from the exome and capture-based sequencing alignments.

Enrichment for c-AID signature amongst *IGLL5* mutations

Five of 40 *IGLL5* variants (in 25 patients) were consistent with a c-AID signature [i.e., mutation of C to T or G at a WRCY motif, where W = A or T, R = purine (G or A) and Y = pyrimidine (C or T)]. We determined the likelihood that this number of c-AID-induced mutations would occur by chance using a binomial test, where the binomial probability was the background probability of such a mutation within the gene. We empirically estimated this probability to be 0.005 by defining it as the product of the following probabilities: (1) the probability (observed frequency within the data) that a four-nucleotide motif within the sequenced region of *IGLL5* is a WRCY (19/632); (2) the probability of mutating the C (not the Y) within this motif (1/4); and (3) the probability (observed frequency within the data) of a C being mutated to either a G or a T (8/11).

Mutual co-occurrence and mutual exclusivity

Mutation co-occurrence and mutual exclusivity were calculated using MuSiC.[17] Raw *p*-values calculated using 100,000 permutations are reported.

IGLL5 survival analysis

Clinical and non-synonymous SNV and indel data were downloaded from the MMRF Researcher Gateway as part of CoMMpass trial IA9 data release (files STAND_ALONE_SURVIVAL.csv and MMRF_CoMMpass_IA9_All_Canonical_NS_Variants.txt). These data were generated as part of the Multiple Myeloma Research Foundation Personalized

Medicine Initiatives (https://research.themmrf.org and www.themmrf.org). Progression events and times were defined using the "ttcpfs" and "censpfs" fields, respectively, from the file STAND_ALONE_SURVIVAL.csv. Survival analysis was performed in R using the survival and survminer packages: Kaplan-Meier curves were generated using survfit and plotted using ggsurvplot, while a Cox proportional hazards model was fit using coxph.

Fluorescence *in situ* hybridization

Fluorescent *in situ* hybridization (FISH) was performed on ACK lysed BM aspirates using cIg-FISH as previously described.[18] All samples were hybridized with commercial probes (Abbott/Vysis). A dual color break apart probeset for 14q32 was first used to determine if there was a translocation involving the IGH locus. If the break apart was positive, a reflex to the most common translocations observed in multiple myeloma were used: t(11;14)(q13;q32) (i.e., *CCND1*/*IGH*), followed by t(4;14)(p16.3;q32) (i.e., *FGFR3*/*IGH*), and then lastly t(14;16)(q32;q23) (i.e., *IGH*/*MAF*).

RNA-seq expression data

RNA-seq expression data from MM samples was obtained from the Multiple Myeloma Research Foundation (MMRF) Researcher Gateway (rna_expr.eligible.gct; research.themmrf.org). A gene was considered expressed in MM and hence eligible for inclusion on the targeted capture panel if it's expression exceeded an FPKM of 0.001 in at least half of the 33 samples in the data set. *DERL3* expression across MM samples was obtained from the interim analysis 7 (IA7) release of the CoMMpass trial, which was also downloaded from the MMRF Research Gateway.

SUPPLEMENTAL FIGURE AND TABLE LEGENDS

Table S1. BED file of capture sequencing platform probes. Coordinates in hg19 genome.

Table S2. Names and Ensembl identifiers of genes targeted by capture sequencing platform.

Figure S1. On-target coverage of tumor samples averages 104X. Total duplicate bases, total aligned off-target bases, total aligned on-target bases, and total unaligned bases (left y axis) of tumor samples (x axis). Percent of on-target bases (right y axis) aligned at 30X coverage.

Figure S2. On-target coverage of normal samples averages 107X. Total duplicate bases, total aligned off-target bases, total aligned on-target bases, and total unaligned bases (left y axis) of normal samples (x axis). Percent of on-target bases (right y axis) aligned at 30X coverage.

Table S3. Targeted regions of chromosome arms. Coordinates (hg19) of each chromosome arm are given by chr, start, and stop columns. Targeted regions are indicated by restrict.start and restrict.end columns. Fraction of the entire chromosome arm that is within targeted region is specified in column frac.of.orig.

Figure S3. Filtering scheme automates detection of CNVs. (A) Background null distribution of (logarithm of) tumor/normal sequencing depth ratios (y axis) within diploid regions is established from a chromosome unlikely to harbor copy-number events across most multiple myeloma samples. Vertical lines represent chromosome boundaries, ordered from 1 to 22

along x axis. (B) Outliers and potential CNVs that perturb the null distribution are separated from the unperturbed/diploid-region ratios by fitting a gaussian mixture model to the (non-log-transformed) depth ratios. (C) Only those regions whose ratios are further than two standard deviations from the mean diploid ratio are assessed for, and ultimately identified as having (blue; $p < 0.05$), CNVs by CopyCat2.

Table S4. Annotated CNVs called by CopyCat2. cna.type: type of CNV (focal, arm, or hyperdiploid); cna.region: affected arm(s) and/or chromosome(s); cna.dir: amplification or deletion; genes: genes encompassed by CNV.

Figure S4. Targeted sequencing identifies arm-level CNVs. Frequency of arm-level amplifications (red) and deletions (blue).

Figure S5. SVM-based filtering reduces false positives in *IGH* translocation calls. Each point represents a LUMPY canonical *IGH* translocation call (including those involving *MYC*) in terms of number of split reads (x axis) and number of discordant paired-end reads (y axis) supporting it. Points are colored according to whether they were validated by FISH (blue) or not (red) and are jittered to improve visibility. If multiple translocations for the same canonical partner were called in a sample, only that with maximal total evidence (number of split reads plus number of paired-end reads) is depicted. Optimal SVM decision hyperplane (at interface of blue and yellow fill) was inferred from canonical *IGH* translocations (excluding those involving *MYC*) in training set.

Table S5: Annotated and SVM-scored LUMPY *IGH* translocation calls. Translocation partner 1 indicated by “chrom” and “pos” and partner 2/mate indicated by “mate_chrom” and “mate_pos” (hg19 coordinates). “sr”: number of split reads supporting translocation; “pe”:

number of paired-end reads supporting translocation; “tot.evidence”: total evidence (i.e., sum of “sr” and “pe”) supporting translocation. “mm.gene1” and “mm.gene2”: multiple myeloma-relevant gene within 1Mb (irrespective of strand/orientation) of breakpoint “pos” or “mate_pos,” respectively. “census.gene1” and “census.gene2”: gene annotated in Cancer Gene Census gene within 1Mb (irrespective of strand/orientation) of breakpoint “pos” or “mate_pos,” respectively. “svm”: output of SVM classifier; 1 indicates likely translocation and 0 indicates likely false positive.

Table S6. Genes involved in canonical *IGH* translocations. LUMPY translocation calls were filtered to be within 1MB up- or down-stream of most extremal genomic (hg19) coordinate of these genes.

Figure S6. Capture sequencing identifiers a complex translocation involving chromosomes 11, 13, and 14. (A) IGV screenshot showing discordant paired-end sequencing reads [in which one end maps to the *IGH* locus (chromosome 14) and the mate maps to either chromosome 11 (green) or chromosome 13 (orange)] or split reads [in which a mate’s bases align to both chromosome 14 (solid color) and partner chromosome (as indicated; rainbow color)]. (B) Circos plot showing regions of chromosomes 11, 13, and 14 involved in putative translocation.

Figure S7. *DERL3* is over-expressed in multiple myeloma relative to other cancer types. Expression values from the Cancer Cell Line Encyclopedia.

Figure S8. **SVM-based filtering reduces false positives in *MYC* translocation calls.** Each point represents a LUMPY (A) intra- or (B) inter-chromosomal *MYC* translocation call (excluding those involving *IGH*) in terms of number of split reads (x axis) and number of discordant paired-

end reads (y axis) supporting it. Points are colored according to whether they were called within a tumor (blue) or normal (red) sample and are jittered to improve visibility. If multiple translocations for the same canonical partner were called in a sample, only that with maximal total evidence (number of split reads plus number of paired-end reads) is depicted. SVM decision hyperplanes (at interface of blue and yellow fill) were fit to filter all putative translocation calls within normal samples.

Table S7. Annotated and SVM-scored LUMPY *MYC* translocation calls. Header as in Table S5.

Table S8. SNVs and indels detected during initial targeted sequencing.

Figure S9. Exome and capture sequencing results are highly correlated. (A) Variant allele frequency (VAF) of variants discovered by both capture and exome sequencing (gray), by capture sequencing only (green), or by exome sequencing only (blue). (B) VAF (y axis) density (x axis) of exome-specific variants. (C) VAF (x axis) density (y axis) of capture sequencing-specific variants.

Figure S10. Downsampling confirms limited impact of additional sequencing depth. Total number of non-silent, non-*IGH* mutations passing filter (y axis) as a function of sequencing depth (x axis) relative to total depth of 15 deeply sequencing samples. Random downsampling of reads and subsequent variant calling was performed five times for each of the indicated percentages.

Table S9. MuSiC analysis of mutation co-occurrence and mutual exclusivity. *P*-values for co-occurrence and mutual exclusivity of mutations involving genes listed in columns Gene1 and Gene2 in columns Pvalue_And and Pvalue_Xor, respectively.

Figure S11. *IGLL5* single nucleotide variants are frequently clonal. Variant allele frequencies (VAFs) of *IGLL5* SNVs in copy number-neutral loci.

REFERENCES

1 Griffith, M. *et al.* Genome Modeling System: A Knowledge Management Platform for Genomics. *PLoS Comput Biol* **11**, e1004274, doi:10.1371/journal.pcbi.1004274 (2015).

2 Sehn, J. K., Abel, H. J. & Duncavage, E. J. Copy number variants in clinical next-generation sequencing data can define the relationship between simultaneous tumors in an individual patient. *Exp Mol Pathol* **97**, 69-73, doi:10.1016/j.yexmp.2014.05.008 (2014).

3 Miller, C. A. *et al.* SciClone: inferring clonal architecture and tracking the spatial and temporal patterns of tumor evolution. *PLoS Comput Biol* **10**, e1003665, doi:10.1371/journal.pcbi.1003665 (2014).

4 Layer, R. M., Chiang, C., Quinlan, A. R. & Hall, I. M. LUMPY: a probabilistic framework for structural variant discovery. *Genome Biol* **15**, R84, doi:10.1186/gb-2014-15-6-r84 (2014).

5 Chiang, C. *et al.* SpeedSeq: ultra-fast personal genome analysis and interpretation. *Nat Methods* **12**, 966-968, doi:10.1038/nmeth.3505 (2015).

6 Li, H. *et al.* The Sequence Alignment/Map format and SAMtools. *Bioinformatics* **25**, 2078-2079, doi:10.1093/bioinformatics/btp352 (2009).

7 Futreal, P. A. *et al.* A census of human cancer genes. *Nat Rev Cancer* **4**, 177-183, doi:10.1038/nrc1299 (2004).

8 Walker, B. A. *et al.* Characterization of IGH locus breakpoints in multiple myeloma indicates a subset of translocations appear to occur in pregerminal center B cells. *Blood* **121**, 3413-3419, doi:10.1182/blood-2012-12-471888 (2013).

9 Sun, Z. J. & Kitchingman, G. R. Sequencing of selected regions of the human immunoglobulin heavy-chain gene locus that completes the sequence from JH through the delta constant region. *DNA Seq* **1**, 347-355 (1991).

10 Mills, F. C., Harindranath, N., Mitchell, M. & Max, E. E. Enhancer complexes located downstream of both human immunoglobulin Calpha genes. *J Exp Med* **186**, 845-858 (1997).

11 Hayday, A. C. *et al.* Activation of a translocated human c-myc gene by an enhancer in the immunoglobulin heavy-chain locus. *Nature* **307**, 334-340 (1984).

12 Li, H. & Durbin, R. Fast and accurate short read alignment with Burrows-Wheeler transform. *Bioinformatics* **25**, 1754-1760, doi:10.1093/bioinformatics/btp324 (2009).

13 Larson, D. E. *et al.* SomaticSniper: identification of somatic point mutations in whole genome sequencing data. *Bioinformatics* **28**, 311-317, doi:10.1093/bioinformatics/btr665 (2012).

14 Cibulskis, K. *et al.* Sensitive detection of somatic point mutations in impure and heterogeneous cancer samples. *Nat Biotechnol* **31**, 213-219, doi:10.1038/nbt.2514 (2013).

15 Saunders, C. T. *et al.* Strelka: accurate somatic small-variant calling from sequenced tumor-normal sample pairs. *Bioinformatics* **28**, 1811-1817, doi:10.1093/bioinformatics/bts271 (2012).

16 Koboldt, D. C. *et al.* VarScan 2: somatic mutation and copy number alteration discovery in cancer by exome sequencing. *Genome Res* **22**, 568-576, doi:10.1101/gr.129684.111 (2012).

17 Dees, N. D. *et al.* MuSiC: identifying mutational significance in cancer genomes. *Genome Res* **22**, 1589-1598, doi:10.1101/gr.134635.111 (2012).

18 Ahmann, G. J. *et al.* A novel three-color, clone-specific fluorescence in situ hybridization procedure for monoclonal gammopathies. *Cancer genetics and cytogenetics* **101**, 7-11 (1998).
