# Supplementary figures and images for: A multiple myeloma-specific capture sequencing platform discovers novel translocations and frequent, risk-associated point mutations in IGLL5
Source: Blood Cancer J. 2018 Mar 21;8(3):35. doi: 10.1038/s41408-018-0062-y (PMC5862875; doi:10.1038/s41408-018-0062-y)

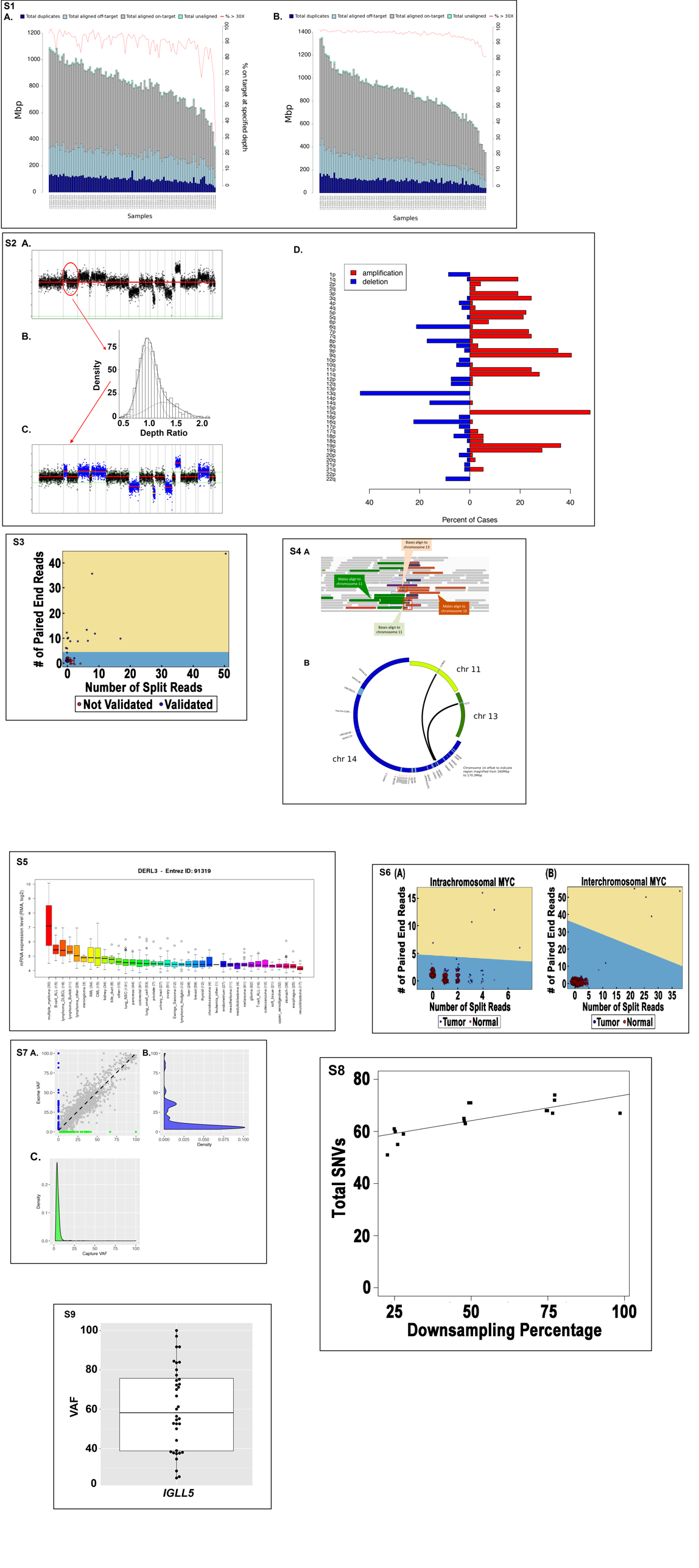

Supplement: Supplementary file 3 — Supplemental Figures(TIF 7876 kb) [file 41408_2018_62_MOESM3_ESM.tif]
